# A new implementation for online calculation of manipulator Jacobian

**DOI:** 10.1371/journal.pone.0212018

**Published:** 2019-02-26

**Authors:** Pramod Chembrammel, Thenkurussi Kesavadas

**Affiliations:** 1 Health Care Engineering Systems Center, University of Illinois at Urbana-Champaign, Urbana, Illinois, United States of America; 2 Industrial and Enterprise Systems Engineering Dept., University of Illinois at Urbana-Champaign, Urbana, Illinois, United States of America; Huazhong University of Science and Technology, CHINA

## Abstract

This paper describes a new implementation for calculating Jacobian and its time derivative for robot manipulators in real-time. The estimation of Jacobian is the key in the real-time implementation of kinematics and dynamics of complex planar or spatial robots with fixed as well as floating axes in which the Jacobian form changes with the structure. The proposed method is suitable for such implementations. The new method is based on matrix differential calculus. Unlike the conventional methods, which are based on screw theory, the Jacobian calculation in the proposed approach has been reduced to the inner product of two matrices. Use of the new method to derive linear and angular velocity parts of Jacobian and its time derivative is described in detail. We have demonstrated the method using a two-DOF spatial robot and a hyper-redundant spatial robot.

## 1 Introduction

The agility of a robot depends on how fast it can adapt to an environment. The dynamics of a robot required for such control primarily involves calculation of Jacobian. It relates joint rates to end-effector velocities and relates end-effector forces to joint torques. Also, the columns of the Jacobian are the instantaneous directions in which a desired point on a robot can move [1]. Thus, Jacobian is the key to the analysis and control of robots. Various methods of calculating the Jacobian with a fixed number of links have been explored and successfully demonstrated in [2–4] and in numerous other publications on robot kinematics and dynamics.

Current methods [2, 5] deal with fixed-configuration of the robot in which the Jacobian is calculated a priori with respect to a fixed set of points on the robot. In some cases, Jacobian needs to be calculated with respect to points that vary within a local coordinate system. Such points arise in the case of fixed as well as reconfigurable robots. In the case of reconfigurable robots, these points are generated because of the change in configuration (eg: change in the configuration of a walking robot or addition of intermediate links to a serial manipulator), whereas in the case of fixed-configuration robots, the variation is either due to floating axes [6] or due to external influences. Such variations require that dynamic control be exercised with respect to the varying points, which demand recursive and real-time calculations. Conventional methods of finding Jacobian fail in such cases, these methods involve manual reformulation of the Jacobian when it changes form for the above reasons. In this paper, we propose a new method for the real-time calculation and reformulation of Jacobian. This method can be applied to fixed as well as reconfigurable robots. The scope of this paper is limited to fixed-configuration. The number of matrix operations is 2*n* + *n*/(2*δ*) (see Section 6) as compared to (3*n* − 6) of the widely used Renaud’s method [5]. The performance of the proposed method is comparable to that of Renaud’s method for low DOF (*n* < 8) robots and is superior for high-DOF robots (see Section 6). In terms of reformulation, Renaud’s method cannot be compared to the proposed method, since Renaud’s method involves manual reformulation if the Jacobian changes form.

Our method also permits the autonomous online computation of the time derivative, $\dot{J}$, of Jacobian, which is required for the robot dynamics as well as to resolve kinematic redundancy using optimal control techniques [7]. $\dot{J}$ is also required for the estimation of link positions from joint angle measurements (proprioception) using optimal estimation techniques like unscented Kalman filters and particle filters [8–10]. Symbolic computation of Jacobian and its derivative is detailed in [11], but it requires extensive manual intervention and hence is not an autonomous approach. In addition, we are not interested in a symbolic derivation, but in a numerical implementation that can autonomously estimate the Jacobian and its time derivative. Authors could not find any available literature on systematic and autonomous derivation of $\dot{J}$.

## 2 Related work on Jacobian estimation

Conventional methods use either the loop closure method [1, 2, 4, 12] or screw theory [2, 5] to calculate Jacobian. The main disadvantage of the loop-closure method is that although it is very useful for planar and spatial mechanisms with a few degrees of freedom (DOF), it is not suitable for complex spatial mechanisms with high DOF and linkages with lower pair joints [13]. Screw-theory-based methods are useful in such cases, but fail to address situations with floating axes of the joints, as demonstrated in [6, 14]. All the reported works efforts have dwelt on finding Jacobian with respect to only one point, i.e., the end-effector. However, there are many situations in which the Jacobian has to be calculated with respect to many points on the robot, which may also vary with time. These methods do not perform automated calculation of the Jacobian matrix and require manual intervention before final solution can be found. That is the main obstacle to real-time implementation.

### 2.1 Comparison of existing methods

This section compares the computational efficiencies of different existing methods based on screw theory. There are six such methods, as discussed in [5]. Although the methods use the same basic concepts, they yield different forms of the Jacobian matrix. The methods work on the premise of finding the angular (*ω*) and linear (**v**) velocities of the desired point on the manipulator by summing the joint rates.

$$\begin{array}{c} \omega =\sum_{i=1}^{n}{\dot{q}}_{i}z_{i-1} \end{array}$$ (1)

$$\begin{array}{c} v=-\sum_{i=1}^{n}\left({\dot{q}}_{i}z_{i-1}\right)\times p_{i-1} \end{array}$$ (2)

where (.) × (.) is a cross product, **p**_*i*−1_ is the coordinate of the desired point in the local coordinate system, and all other **p**_*i*_ represent the coordinates of the origins of the link coordinate systems with respect to the preceding coordinate system. These methods differ only in the way the coefficients are extracted out of Eqs (1) and (2) to form the Jacobian. Among the methods discussed in [5], the first three approaches differ from the fourth and fifth in that their computations begin from opposite ends of a multi-link robot. The first three methods begin from the base, while the others begin from the end-effector. Among them, the fourth method, developed by Renaud [15], is computationally superior to the other methods with (30*n* − 87) multiplications, (15*n* − 66) additions and subtractions, and (2*n* − 2) sines and cosines. Here, *n* is the number of degrees of freedom. The number of matrix operations (3*n* − 6) is also the least for this method [5]. Although these methods are very efficient in calculating the Jacobian, they require manual intervention to select the axis of rotation or translation (**z**_*i*−1_ in Eq (1)), depending on the type of joint variable. That renders the existing methods incapable of finding the form of Jacobian in real-time, both for re-configurable and for fixed-configuration robots in which the Jacobian with respect to a new point has to be found.

Automatic estimation of Jacobian via zeroing dynamics [16] while the kinematic structure (DH parameters) of a robot is not known, is explained in [17]. In this method, the Jacobian is iteratively estimated by tracking the kinematics of the end-effector using feedback from external sensors like cameras or inertial measurement units (IMUs). However, external feedback makes this approach less attractive for most of the applications for which external tracking is either not required or not possible. In addition, this method is not applicable for the regular (nonredundant) robots; it is applicable only for redundant robots.

In this paper, we propose a new method that facilitates estimation of Jacobian in real-time. Like the loop-closure method, the proposed method is founded on matrix differential calculus; however, the procedure involved in finding the Jacobian is different from those of the loop-closure and screw-theory-based methods. Also, it does not distinguish between joints, either prismatic or revolute. It is therefore possible to use numerical methods for the real-time estimation of Jacobian.

This paper is laid out as following. In section 3, proposed method is described. In section 4, we discuss how we demonstrated it using a simple two-link planar manipulator. The method is summarized as an algorithm in section 5. In section 6 we explain how we demonstrated the real-time application of the method with the help of a redundant spatial robotic arm.

## 3 The new method

A point, **p**, in a coordinate system, *j*, attached to a link of a robot is represented in another coordinate frame, *i*, or the base coordinate frame, through the use of homogeneous transformation.

$$\begin{array}{c} x^{i}\left(q\right)=A\left(q\right)p^{j} \end{array}$$ (3)

where **A** is the homogeneous transformation matrix, and **x**^*i*^ is the point after transformation. The superscripts represent the respective coordinate systems. The matrix **A** is a function of the generalized coordinates, **q**, of the robot thus making **x**^*i*^ also a function of **q**.

The velocity of the transformed point in the *i*^th^ coordinate system is given by

$$\begin{array}{c} {\dot{X}}^{i}\left(q\right)=J\left(q,p\right)\dot{q} \end{array}$$ (4)

where **J** is the Jacobian of the robot with respect to the point $p^{j}.\;{\dot{X}}^{i}$ has both global linear velocity, ($v^{i}={\dot{x}}^{i}$), and angular velocity, *ω^i^*. As is evident from Eq (4), the Jacobian relates the joint rates to the velocities of the desired point **p**^*j*^ represented in frame *i*. In the transformation of Eqs (3) to (4), the coordinates of the point **p** in the coordinate system *j* have been absorbed into the **J**. Thus, if the Jacobian is proved to be a function of independent entities, i.e. as a function of the derivative of the transformation matrix [18] **A**(**q**) and the constant vector **p**, then it is only sufficient to perform simple matrix multiplications to find out the Jacobian. This is the key to real-time estimation of **J**(**q**).

**J** has two parts; a relative velocity part of dimension (3 × *n*), and an angular velocity part of dimension (3 × *n*) [2]. The following subsections explain the real-time estimation of both parts of **J**. (It is to be noted that **x** and *ω* are represented in *i* and that **p** is represented in *j*. Hence, superscripts are ignored in the following sections for conciseness).

### 3.1 Calculation of linear velocity part

The velocity equation in Eq (4) is derived as follows. Taking the time derivative of Eq (3),

$$\begin{array}{c} \dot{x}\left(q\right)=\dot{A}\left(q\right)p \end{array}$$ (5)

The time derivative of **p** is not present in Eq (5) since it is a constant vector in the local coordinate system, *j*. The time derivative of **A**(**q**) is written as

$$\begin{array}{c} \dot{A}\left(q\right)=\sum_{i=1}^{n}\frac{\partial A(q_{i})}{\partial q_{i}}{\dot{q}}_{i} \end{array}$$ (6)

Using Eq (6) in Eq (5),

$$\begin{array}{c} \dot{x}\left(q\right)=[\sum_{i=1}^{n}\frac{\partial A(q_{i})}{\partial q_{i}}{\dot{q}}_{i}]p \end{array}$$ (7)

Since the vector **p** is common and ${\dot{q}}_{i}$ is a scalar, Eq (7) is rewritten as

$$\begin{array}{c} \dot{x}\left(q\right)=\sum_{i=1}^{n}[\frac{\partial A(q_{i})}{\partial q_{i}}p]{\dot{q}}_{i} \end{array}$$ (8)

In matrix notation, Eq (8) is represented by

$$\begin{array}{c} \dot{x}\left(q\right)=J_{v}\left(q\right)\dot{q} \end{array}$$ (9)

where, **J**_*v*_(**q**) is the linear velocity part of **J**(**q**). What is left to perform is to decompose **J**_*v*_(**q**) as a product of independent matrices. From Eq (8), **J**_*v*_(**q**) is given by

$$\begin{array}{c} J_{v}\left(q\right)=[\frac{\partial A(q_{1})}{\partial q_{1}}p\;...\;\frac{\partial A(q_{i})}{\partial q_{i}}p\;...\;\frac{\partial A(q_{n})}{\partial q_{n}}p] \end{array}$$ (10)

Eq (10) is written as the product of two matrices:

$$\begin{array}{c} J_{v}\left(q\right)=LP \end{array}$$ (11)

where

$$\begin{array}{c} L=[\frac{\partial A(q_{1})}{\partial q_{1}}\;...\frac{\partial A(q_{i})}{\partial q_{i}}\;...\;\frac{\partial A(q_{n})}{\partial q_{n}}] \end{array}$$ (12)

and **P** = **I**_*n*_ ⊗ **p**, where **I**_*n*_ is an (*n* × *n*) identity matrix, **p** is in homogeneous form, and (.) ⊗ (.) is the Kronecker product.

$$\begin{array}{c} p={\left[x\;y\;z\;1\right]}^{T} \end{array}$$ (13)

The dimensions of **J**_*v*_, **L**, and **P** are, respectively, (4 × *n*), (4 × 4*n*), and (4*n* × *n*). Thus, the linear velocity part of the Jacobian is

$$\begin{array}{c} J_{v}\left(q\right)={\left(LP\right)}_{\left(1-3,1-n\right)} \end{array}$$ (14)

It is to be noted that **P** is a sparse matrix and hence can efficiently store values.

### 3.2 Calculation of angular velocity part

The matrix **A** in Eq (3) is also called the *transition matrix* from the initial value to the final value [2, 19], and is written as

$$\begin{array}{c} x\left(q,t\right)=A(q(t))x\left(q,0\right) \end{array}$$ (15)

Taking the time derivative of Eq (15),

$$\begin{array}{c} \dot{x}\left(q,t\right)=\dot{A}(q(t))x\left(q,0\right) \end{array}$$ (16)

Since **A** is invertible, from Eq (15),

$$\begin{array}{c} \dot{x}\left(q,t\right)=\dot{A}(q(t))A(q(t){)}^{-1}x\left(q,t\right) \end{array}$$ (17)

But,

$$\begin{array}{c} \dot{A}(q(t))A((q(t){)}^{-1}=[\begin{array}{cc} \dot{R} & {\dot{r}}^{T}\\ 0 & 0 \end{array}]\;[\begin{array}{cc} R^{T} & -R^{T}r^{T}\\ 0 & 1 \end{array}] \end{array}$$ (18)

where **R** is the (3 × 3) orientation part of **A**, and **r**^T^ is a (3 × 1) translation part of **A**. Eq (18) is written as

$$\begin{array}{c} \dot{A}(q(t))A(q(t){)}^{-1}=[\begin{array}{cc} \dot{R}R^{T} & {\dot{r}}^{T}-\dot{R}R^{T}r^{T}\\ 0 & 0 \end{array}] \end{array}$$ (19)

The element $\dot{R}R^{\text{T}}$ of Eq (19) is called the *twist matrix* [2] which is the skew-symmetric form of the angular velocity vector [20] of the desired link. Similar to Eq (8), the left hand side of Eq (19) is written using (6) as

$$\begin{array}{c} \dot{A}(q(t))A(q(t){)}^{-1}=\sum_{i=1}^{n}[\frac{\partial A(q_{i})}{\partial q_{i}}A(q(t){)}^{-1}]{\dot{q}}_{i} \end{array}$$ (20)

The right hand side of Eq (20) is thus written as

$$\begin{array}{c} \dot{A}(q(t))A(q(t){)}^{-1}=\sum_{i=1}^{n}L_{i}P{\dot{q}}_{i} \end{array}$$ (21)

where **L** is given by Eq (12) and **P** ≜ **A**^−1^. The subscript *i* on the right hand side represents the *i*^th^ (4 × 4) block of the matrix **L**. The first (3 × 3) matrix of (**L**_*i*_**P**) represents the twist matrix for each link. Thus, the angular velocity part of the Jacobian is

$$\begin{array}{c} J_{\omega ,i}=[{\left(L_{i}P\right)}_{\left(3,2\right)}\;{\left(L_{i}P\right)}_{\left(1,3\right)}\;{\left(L_{i}P\right)}_{\left(2,1\right)}{]}^{\text{T}} \end{array}$$ (22)

The subscript *i* on the left hand side of Eq (22) represents the column index of the manipulator Jacobian. The subscripts (3, 2), (1, 3), and (2, 1) of the twist matrix from (**L**_*i*_**P**) represent the *x*, *y*, *z* components of the angular velocity part of the Jacobian. To use the standard notation, the complete manipulator Jacobian is thus

$$\begin{array}{c} J=[\begin{array}{c} J_{v}\\ J_{\omega } \end{array}] \end{array}$$ (23)

From Eqs (12), (14), and (22), it is observed that the Jacobian is affected by any variation in the point **p**. With this method. unlike the conventional methods, this variation can be easily incorporated into the calculation of the Jacobian just by recalculating **P** = **I**_*n*_ ⊗ **p**. This method has applications in fixed-configuration robots with varying points.

### 3.3 Time derivative, $\dot{\text{J}}$, of Jacobian

In order to find out the time derivative of Jacobian, we consider the linear and velocity parts separately because **P** is different in the two cases. The total time derivative of the linear part is

$$\begin{array}{c} {\dot{J}}_{v}=\sum_{i=1}^{n}\frac{\partial J_{v}}{\partial q_{i}}{\dot{q}}_{i} \end{array}$$ (24)

Since **P** is a constant from Eq (13),

$$\begin{array}{c} \frac{\partial J_{v}}{\partial q_{i}}=\frac{\partial L}{\partial q_{i}}P \end{array}$$ (25)

Similarly, the total time derivative of the angular part is

$$\begin{array}{c} {\dot{J}}_{\omega }=\sum_{i=1}^{n}\frac{\partial J_{\omega }}{\partial q_{i}}{\dot{q}}_{i} \end{array}$$ (26)

Using Eq (22),

$$\begin{array}{c} \frac{\partial J_{\omega ,i}}{\partial q_{j}}=\frac{\partial }{\partial q_{j}}([{\left(L_{i}P\right)}_{\left(3,2\right)}\;{\left(L_{i}P\right)}_{\left(1,3\right)}\;{\left(L_{i}P\right)}_{\left(2,1\right)}{]}^{T}) \end{array}$$ (27)

From Eq (21), **P**, in this case, is not a constant. Therefore,

$$\begin{array}{c} \frac{\partial (L_{i}P)}{\partial q_{j}}=\frac{\partial L_{i}}{\partial q_{j}}P+L_{i}\frac{\partial P}{\partial q_{j}} \end{array}$$ (28)

But,

$$\begin{array}{c} \frac{\partial P}{\partial q_{j}}=-A^{-1}\frac{\partial A}{\partial q_{j}}A^{-1} \end{array}$$ (29)

where, **L**_*i*_ is the *i*^th^ (4 × 4) block of **L**. Eq (29) is a standard matrix relationship [21]. By using Eq (29) in Eq (28), we obtain

$$\begin{array}{c} \frac{\partial (L_{i}P)}{\partial q_{j}}=(\frac{\partial L_{i}}{\partial q_{j}}-L_{i}A^{-1}L_{j})A^{-1} \end{array}$$ (30)

## 4 Demonstration

**Fig 1:**
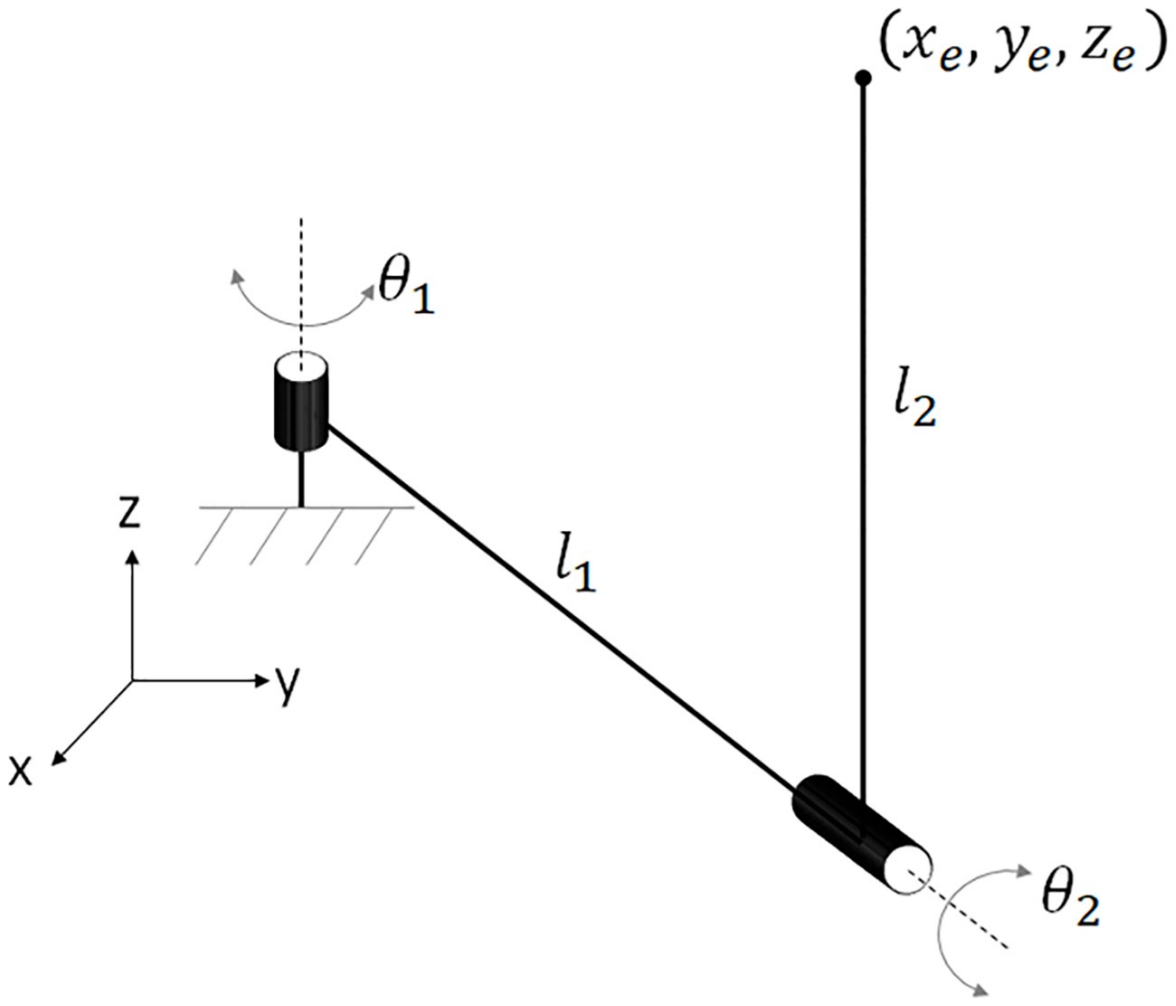
Two-link spatial robot for demonstration of the method.

In this section, the concepts discussed in the previous section are demonstrated using a two-DOF spatial manipulator shown in Fig 1.

Considering an arbitrary point (*x*_*e*_, *y*_*e*_, *z*_*e*_) in the local coordinate frame attached to the second link:

$$\begin{array}{c} x\left(q\right)=[\begin{array}{cccc} -S\theta_{1}C\theta_{2} & C\theta_{1} & -S\theta_{1}S\theta_{2} & l_{1}C\theta_{1}-l_{2}S\theta_{1}C\theta_{2}\\ C\theta_{1}C\theta_{2} & S\theta_{1} & C\theta_{1}S\theta_{2} & l_{1}S\theta_{1}+l_{2}C\theta_{1}C\theta_{2}\\ S\theta_{2} & 0 & -C\theta_{2} & l_{2}S\theta_{2}\\ 0 & 0 & 0 & 1 \end{array}]\;[\begin{array}{c} x_{e}\\ y_{e}\\ z_{e}\\ 1 \end{array}] \end{array}$$ (31)

where *C* stands for *cosine* and *S* for *sine*. The transformation matrix in the above equation is recursively calculated using a homogeneous transformation matrix [18]. Taking the time derivative of Eq (31),

$$\begin{array}{c} \dot{x}\left(q,\dot{q}\right)=\tilde{J}X_{e} \end{array}$$ (32)

where

$$\begin{array}{c} \tilde{J}=J_{1}{\dot{q}}_{1}+J_{2}{\dot{q}}_{2} \end{array}$$ (33)

$$\begin{array}{c} X_{e}=[x_{e}\;y_{e}\;z_{e}\;1{]}^{\text{T}} \end{array}$$ (34)

$$\begin{array}{c} J_{1}=[\begin{array}{cccc} -C\theta_{1}C\theta_{2} & -S\theta_{1} & -C\theta_{1}S\theta_{2} & -l_{1}S\theta_{1}-l_{2}S\theta_{1}C\theta_{2}\\ -S\theta_{1}C\theta_{2} & C\theta_{1} & -S\theta_{1}S\theta_{2} & l_{1}C\theta_{1}-l_{2}S\theta_{1}C\theta_{2}\\ 0 & 0 & 0 & 0\\ 0 & 0 & 0 & 0 \end{array}] \end{array}$$ (35)

and

$$\begin{array}{c} J_{2}=[\begin{array}{cccc} S\theta_{1}S\theta_{2} & 0 & -S\theta_{1}C\theta_{2} & l_{2}S\theta_{1}S\theta_{2}\\ -C\theta_{1}S\theta_{2} & 0 & C\theta_{1}C\theta_{2} & -l_{2}C\theta_{1}S\theta_{2}\\ C\theta_{2} & 0 & S\theta_{2} & l_{2}C\theta_{2}\\ 0 & 0 & 0 & 0 \end{array}] \end{array}$$ (36)

Expanding Eq (32) using Eq (33),

$$\begin{array}{c} \dot{x}\left(q,\dot{q}\right)=[J_{1}{\dot{q}}_{1}+J_{2}{\dot{q}}_{2}]X_{e} \end{array}$$ (37)

Since **X**_*e*_ is common, rearranging Eq (37),

$$\begin{array}{c} \dot{x}\left(q,\dot{q}\right)=J_{1}{\dot{q}}_{1}X_{e}+J_{2}{\dot{q}}_{2}X_{e} \end{array}$$ (38)

In the matrix form,

$$\begin{array}{c} \dot{x}\left(q,\dot{q}\right)=[\begin{array}{cc} J_{1}X_{e} & J_{2}X_{e} \end{array}]\;[\begin{array}{c} {\dot{q}}_{1}\\ {\dot{q}}_{2} \end{array}] \end{array}$$ (39)

In Eq (39), **X**_*e*_ can be factored out to obtain

$$\begin{array}{c} \dot{x}\left(q,\dot{q}\right)=[\begin{array}{cc} J_{1} & J_{2} \end{array}]\;(I_{2}⊗X_{e})\;[\begin{array}{c} {\dot{q}}_{1}\\ {\dot{q}}_{2} \end{array}] \end{array}$$ (40)

If we compare Eq (40) with the standard form of the velocity equation, $\dot{x}=J_{v}\dot{q}$, the linear velocity part of the Jacobian for the two-link manipulator is,

$$\begin{array}{c} J_{v}=[\begin{array}{cc} J_{1} & J_{2} \end{array}]\;(I_{2}⊗X_{e}) \end{array}$$ (41)

Eq (41) is the same as Eq (11), in which **L** = [**J**_1_ **J**_2_] and **P** = (**I**_2_ ⊗ **X**_*e*_). If the end-effector is considered the point of interest, then **X**_*e*_ = [0 0 0 1]^T^. Thus, **J**_*v*_ for the two-DOF spatial manipulator is written as

$$\begin{array}{c} J_{v}=[\begin{array}{cc} -l_{1}S\theta_{1}-l_{2}C\theta_{1}C\theta_{2} & l_{2}S\theta_{1}S\theta_{2}\\ l_{1}C\theta_{1}-l_{2}S\theta_{1}C\theta_{2} & -l_{2}C\theta_{1}S\theta_{2}\\ 0 & l_{2}C\theta_{2}\\ 0 & 0 \end{array}] \end{array}$$ (42)

As given in Eq (14), the first three rows represent the linear part of the Jacobian. Therefore,

$$\begin{array}{c} J_{v}=[\begin{array}{cc} -l_{1}S\theta_{1}-l_{2}C\theta_{1}C\theta_{2} & l_{2}S\theta_{1}S\theta_{2}\\ l_{1}C\theta_{1}-l_{2}S\theta_{1}C\theta_{2} & -l_{2}C\theta_{1}S\theta_{2}\\ 0 & l_{2}C\theta_{2} \end{array}] \end{array}$$ (43)

The angular velocity part is obtained as follows. **P** is

$$\begin{array}{c} P=A^{-1}=[\begin{array}{cccc} -S\theta_{1}C\theta_{2} & C\theta_{1}C\theta_{2} & S\theta_{2} & -l_{2}\\ C\theta_{1} & S\theta_{1} & 0 & -l_{1}\\ -S\theta_{1}S\theta_{2} & C\theta_{1}S\theta_{2} & -C\theta_{2} & 0\\ 0 & 0 & 0 & 1 \end{array}] \end{array}$$ (44)

Using Eqs (35) & (36), and following the expression in Eq (22), the block matrix multiplication (**LP**) will yield,

$$\begin{array}{c} J_{\omega }=[\begin{array}{cc} 0 & C\theta_{1}\\ 0 & S\theta_{1}\\ 1 & 0 \end{array}] \end{array}$$ (45)

From Eqs (43) and (45), the complete Jacobian is

$$\begin{array}{c} J=[\begin{array}{cc} -l_{1}S\theta_{1}-l_{2}C\theta_{1}C\theta_{2} & l_{2}S\theta_{1}S\theta_{2}\\ l_{1}C\theta_{1}-l_{2}S\theta_{1}C\theta_{2} & -l_{2}C\theta_{1}S\theta_{2}\\ 0 & l_{2}C\theta_{2}\\ 0 & C\theta_{1}\\ 0 & S\theta_{1}\\ 1 & 0 \end{array}] \end{array}$$ (46)

Equation Eq (46) is a standard result. The time derivative $\dot{\text{J}}$ is found as follows.

$$\begin{array}{c} {\dot{J}}_{v}=(\frac{\partial L}{\partial \theta_{1}}P){\dot{\theta }}_{1}+(\frac{\partial L}{\partial \theta_{2}}P){\dot{\theta }}_{2} \end{array}$$ (47)

where

$$\begin{array}{c} \frac{\partial L}{\partial \theta_{1}}P=[\begin{array}{cc} -l_{1}C\theta_{1}+l_{2}S\theta_{1}C\theta_{2} & l_{2}C\theta_{1}S\theta_{2}\\ -l_{1}S\theta_{1}-l_{2}C\theta_{1}C\theta_{2} & l_{2}S\theta_{1}S\theta_{2}\\ 0 & 0 \end{array}] \end{array}$$ (48)

$$\begin{array}{c} \frac{\partial L}{\partial \theta_{2}}P=[\begin{array}{cc} l_{2}C\theta_{1}S\theta_{2} & l_{2}S\theta_{1}C\theta_{2}\\ l_{2}S\theta_{1}S\theta_{2} & -l_{2}C\theta_{1}C\theta_{2}\\ l_{2}C\theta_{2} & -l_{2}S\theta_{2} \end{array}] \end{array}$$ (49)

Using Eq (30),

$$\begin{array}{c} \frac{\partial L_{1}P}{\partial \theta_{1}}=\frac{\partial L_{1}P}{\partial \theta_{2}}=\frac{\partial L_{2}P}{\partial \theta_{2}}=0_{4\times 4} \end{array}$$ (50)

And,

$$\begin{array}{c} \frac{\partial L_{2}P}{\partial \theta_{1}}=[\begin{array}{cccc} 0 & 0 & C\theta_{1} & 0\\ 0 & 0 & S\theta_{1} & 0\\ -C\theta_{1} & -S\theta_{1} & 0 & 0\\ 0 & 0 & 0 & 0 \end{array}] \end{array}$$ (51)

Following Eq (27),

$$\begin{array}{c} \frac{\partial J_{\omega }}{\partial \theta_{1}}=[\begin{array}{cc} 0 & -S\theta_{1}\\ 0 & C\theta_{1}\\ 0 & 0 \end{array}],\;\frac{\partial J_{\omega }}{\partial \theta_{2}}=[\begin{array}{cc} 0 & 0\\ 0 & 0\\ 0 & 0 \end{array}] \end{array}$$ (52)

Using Eq (26),

$$\begin{array}{c} {\dot{J}}_{\omega }=[\begin{array}{cc} 0 & -S\theta_{1}\\ 0 & C\theta_{1}\\ 0 & 0 \end{array}]{\dot{\theta }}_{1} \end{array}$$ (53)

The time derivative of the complete Jacobian is,

$$\begin{array}{c} \dot{J}=[\begin{array}{c} {\dot{J}}_{v}\\ {\dot{J}}_{\omega } \end{array}] \end{array}$$ (54)

For a two-DOF robot the formulation may appear to involve more steps than a conventional method. Computationally, there is no clear advantage in using this method for two- or three-link robots (see section 6). However, the expressions in Eqs (35), (36) and (41) can be autonomously calculated using differential calculus. That procedure is also suitable for the real-time calculation of Jacobian with respect to a point other than the chosen point at the end-effector. Also, it is a very useful method for mechanisms with floating axes and robots with high DOF. In prior work, we have very effectively used this method for floating axes to model the dynamics of lower limbs (*n* = 21) in human walking [14], as well as for modelling flexible guide-wire dynamics [22]. The application for high-DOF robots is explained in section 6 with respect to the real-time implementation of a real 7-DOF spatial robot.

## 5 Algorithm to find Jacobian

The method discussed in the previous sections can be summarized in the form of an algorithm.

1. Find the final transformation matrix, **A**.
2. Find the (4 × 4) block matrix,
  $$\begin{array}{c} L_{i}=\frac{\partial A(q)}{\partial q_{i}} \end{array}$$
3. Form the (4 × 4*n*) matrix **L** as given in Eq (12).
4. Find matrix **P** as follows:
  - for the linear part, use **P** = **I**_*n*_ ⊗ **p**.
  - for the angular velocity part, use **P** = **A**^−1^.
5. Calculate the Jacobian matrices **J**_*v*/*ω*_ = **LP** for both the parts.
6. Find the derivative of **L** as $\frac{\partial \text{L}}{\partial q_{i}}$.
7. Calculate the time derivative of the linear part of the Jacobian using Eqs (24) and (25) with **P** = **I**_*n*_ ⊗ **p**.
8. Calculate the time derivative of the angular part of the Jacobian using Eqs (30), (27) and (26) with **P** = **A**^−1^.

From the algorithm and the demonstration in section 4, it is evident that no manual intervention is required. The calculations of matrices **A**, **P** and **L** and their derivatives are autonomously carried out. Hence this method is suitable for real-time implementation for any desired point on the robot.

## 6 Application to high-DOF robots and real-time implementation

**Fig 2:**
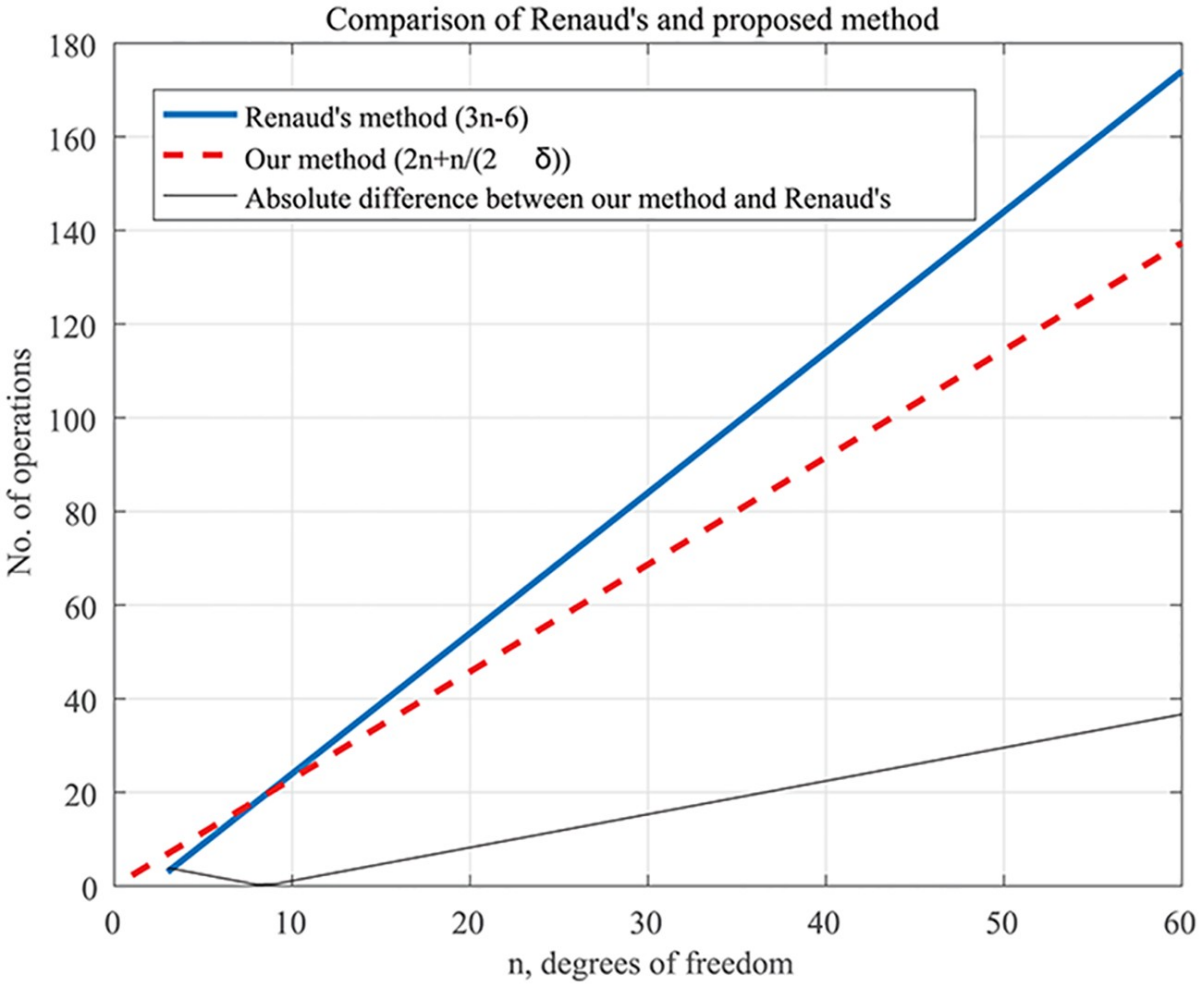
Comparison of computational efficiencies in terms of number of matrix operations in Renaud’s and the proposed method. For (*n* ≥ 6) the number of matrix operations is lower in the proposed method.

The aim of this section is to demonstrate the application to higher-DOF robots for real-time implementation. Following the results given in [5], only the computations that are performed after the computation of the final homogenous transformation matrix, **A**, are considered. The number of matrix operations required is 2*n*, which includes matrix-to-vector and matrix-to-matrix multiplications. The matrices and vectors are in the homogenous form (4 × 4) and (4 × 1). A comparison of the computational efficiency of the proposed method with that of Renaud’s method is given in Table 1 and Fig 2. We chose to compare our method only to Renaud’s method because Renuad’s performance is superior to that of any other existing methods. Renaud’s method involves multiplication of (3 × 3) matrices, so our method will involve 37*n* more scalar multiplications. Thus, the number of operations is modified to 2*n* + *n*/(2*δ*), where *δ* = 37/64 is the ratio between the difference and the number of scalar multiplications in our method. From Fig 2, the performance of our proposed method is comparable to Renaud’s for (*n* ≤ 8) and superior to it for (*n* > 8). Note that there is no definite way of comparing the performance for *n* < 3) since the expression of Renaud’s method is valid only for (*n* ≥ 3).

**Table 1:** Comparison of Renaud’s method and the proposed method.

Method	No. of matrix multiplications	
	(3 × 3)	(4 × 4)
Renaud	3*n*-6	–
Proposed method	–	2*n* + *n*/(2*δ*)

**Fig 3:**
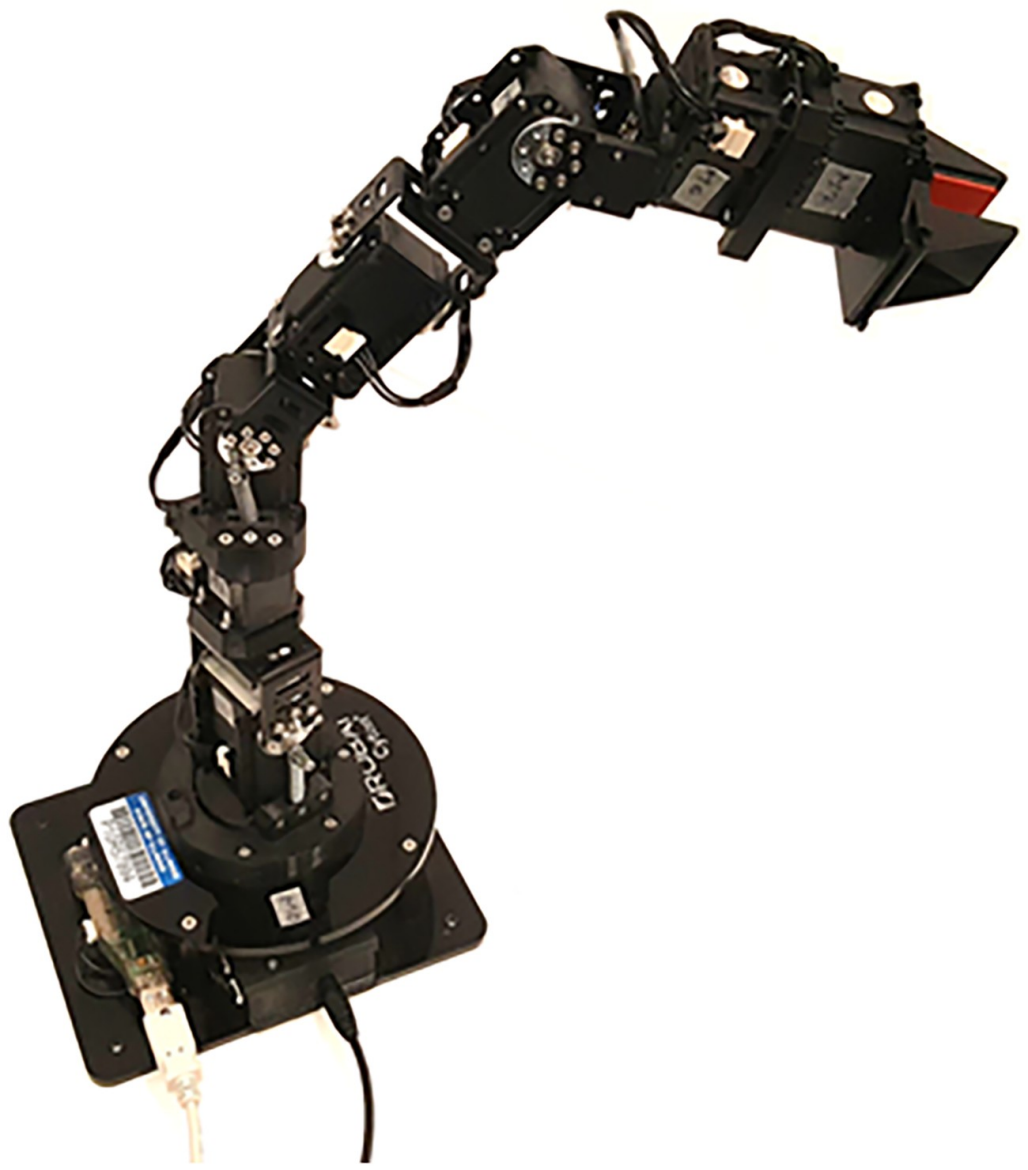
Robai Cyton 7-DOF spatial robot.

**Fig 4:**
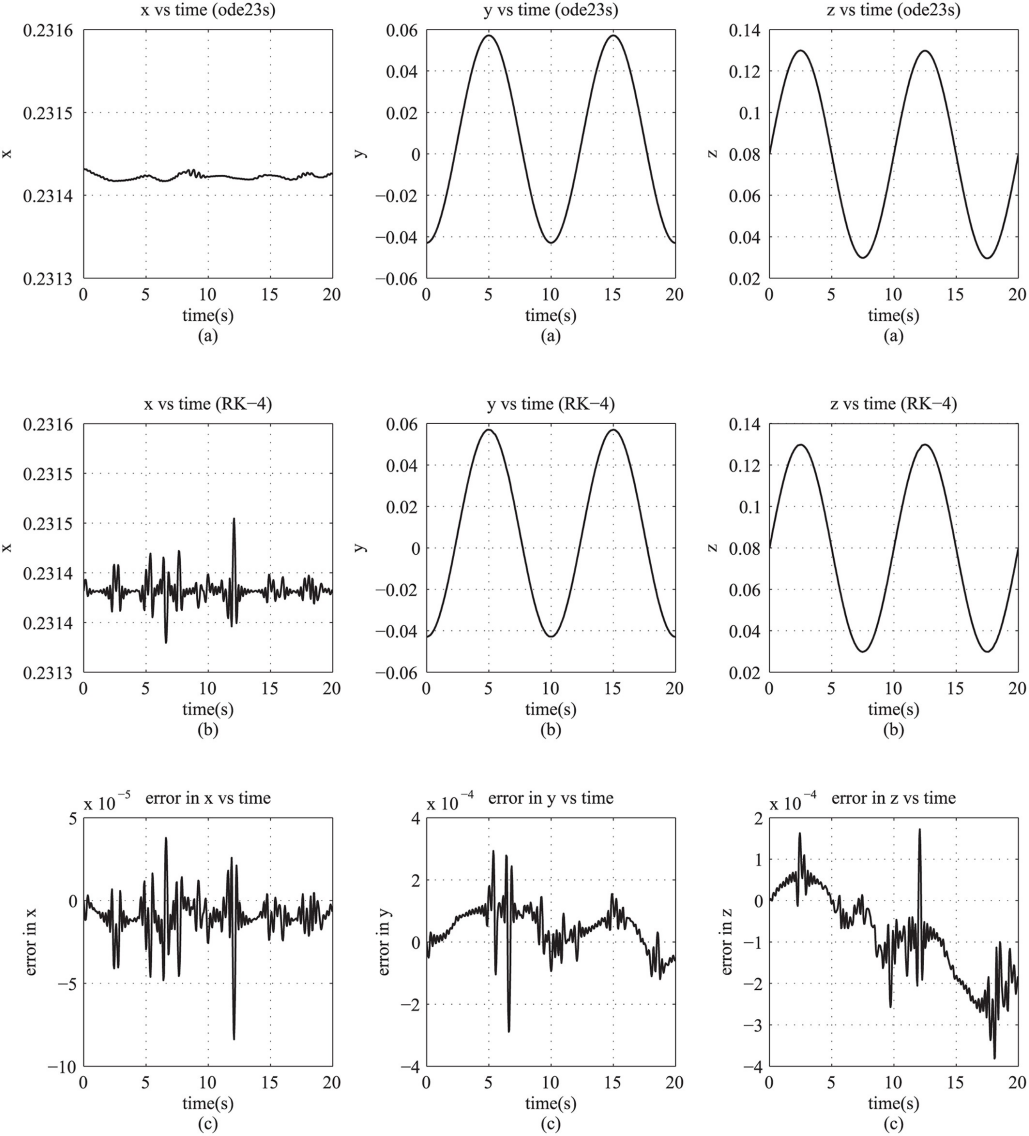
(row-1-(a)) x/y/z components of end-effector trajectory from simulation results using ode23s (Bogacki-Shampine method), (row-2-(b)) x/y/z components of end-effector trajectory from real-time physical implementation using RK-4, and (row-3-(c)) error in x/y/z using Runge-Kutta-4 for integration in real-time implementation.

We demonstrated the real-time implementation using a 7-DOF redundant spatial manipulator (Robai Cyton Gamm-300) (Fig 3). We used Jacobian in the redundancy resolution of the manipulator by using an optimal control technique based on Hamiltonian formulation [7] for natural boundary conditions. That involves estimation of $\dot{\text{J}}$. In our implementation, the end-effector of the manipulator is required to follow a circular trajectory with a 0.05 m radius and a frequency of 0.2 rad/s in the y-z plane at a distance of 0.2314 m from the origin along the x-axis. We implemented the differentiations required to find the Jacobian (as in Eq (10)) by using numerical differential based on the central difference method. The differential equations of the motion of the manipulator are numerically integrated using Runge-Kutta fourth order method. The real-time hardware implementation is done by modifying and appending to the open source C++ libraries for matrix operations, TNT and JAMA [23]. The measurments taken by the joint encoders were simultaneously recorded. We compared the results (i.e., the encoder measurements) (see Fig 4) with the simulation results using a second order semi-implicit integrator (ode23s), in MATLAB; ode23s is an implementation of the Bogacki-Shampine method [24]. An error tolerance of 10^−9^ was used in the integrator. We observed that the hardware implementation and the simulation results matched, as is evident from the negigible errors (see third row of Fig 4). Thus, our approach is suitable for real-time implementation and causes negigible delay in the automatic formulation and estimation of the Jacobian.

## 7 Conclusion

A new implementation for online calculation of manipulator Jaobian has been presented and demonstrated. The method, based on matrix differential calculus, offers a systematic approach to calculating the Jacobian of robotic manipulators. Relative to the conventional methods, the calculation of Jacobian has been reduced to the inner product of two matrices. The matrix differentiations are performed using numerical methods. A real-time implementation of the Jacobian has been demonstrated using a planar two-link robot and a 7-DOF spatial robot. The errors in the hardware implementation of this method have been found to be negligible. Although it is computationally superior only for higher-DOF robots, the method is suitable for autonomous and real-time Jacobian estimations for robots with variable points. Our method is also well-suited for reconfigurable robots, which will be addressed in our future work.

## Supporting information

S1 File Supporting information. This is the data file in .xls format. The file has 5 sheets. The description of each sheet is given in S2 File.
(XLSX)

S2 File Supporting information. This file is in .txt format. This file has the detailed description of the data in S1 File.
(TXT)
